# Detecting sleep and physical activity changes across the perinatal period using wearable technology

**DOI:** 10.1186/s12884-024-06991-1

**Published:** 2024-11-26

**Authors:** Elizabeth A. Claydon, Christa L. Lilly, Erin D. Caswell, Dawna C. Quinn, Shon P. Rowan

**Affiliations:** 1 Department of Social & Behavioral Sciences, West Virginia University School of Public Health, 64 Medical Center Drive, P.O. Box 9190, Morgantown, WV 26505 USA; 2 Department of Epidemiology & Biostatistics, West Virginia University School of Public Health, Morgantown, WV USA; 3 Department of Obstetrics & Gynecology, Baptist Memorial Hospital, Memphis, TN USA; 4 Department of Obstetrics & Gynecology, West Virginia University School of Medicine, Morgantown, WV USA

**Keywords:** Pregnancy, Maternal health, Sleep, Exercise, Wearable technology

## Abstract

**Background:**

Pregnant women may not experience disruptions in sleep duration throughout the course of pregnancy, however, their sleep quality is dramatically impaired. Sleep quality deteriorates throughout pregnancy, reaching its lowest in the third trimester. The purpose of this study was to understand the change in sleep patterns across the perinatal period, as well as the impact of physical activity on sleep.

**Methods:**

A total of 18 physically active women trying to conceive wore a WHOOP strap (a fitness monitor) across the perinatal period. Daily behavior changes were tracked including time awake, hours in deep sleep, physical activity, and time in moderate to vigorous physical activity.

**Results:**

Women maintained overall physical activity levels during and after pregnancy and averaged 20.70 min of physical activity and 6.97 h of sleep per day. Total time in awake hours increased postpartum. Moderate-vigorous physical activity minutes improved deep sleep hours overall (Est. = 0.003 h, *p* < 0.0001) and during pregnancy (Est. = 0.00001 h, *p* = 0.0004). Similar effects were found for all activity minutes, although in post pregnancy the moderating impact of activity minutes no longer maintained significance (*p* = 0.09).\.

**Conclusions:**

Wearable technology, including fitness monitors such as WHOOP straps offer a convenient and less invasive way to track sleep and physical activity during the perinatal period. The findings of this study indicate a positive connection between sleep and engaging in moderate to vigorous activity and any activity throughout the perinatal period. These results may help inform clinical and practical recommendations for physical activity to improve sleep outcomes for pregnant women.

## Plain Language Summary

**Current Knowledge/Study rational:**

There is existing evidence to show that sleep quality declines throughout pregnancy. However, a study continuously gathering sleep data during pregnancy and after delivery has not been conducted. Additionally, less is known about how physical activity during this time may affect sleep quality.

**Study impact:**

This study followed women throughout and after their pregnancy, using wearable technology to track sleep and physical activity during this time. The findings show that physical activity regardless of intensity is associated with a positive impact on sleep. Clinical recommendations regarding physical activity could potentially help improve the lives and sleep of women during this time.

## Introduction

In a society with hundreds of remedies to address poor sleep, an area of the lifespan has been often ignored by these, even though lack of quality sleep at this time may have the most lasting implications. Pregnancy and the post-partum period are times of significant physiological change, which can drastically impact sleep. Although pregnant women may be sleeping a similar or greater amount to non-pregnant women, the quality of that sleep may be substantially impacted, especially as they near their delivery date [1–3]. Common self-reported reasons for poor sleep include frequent urination [4], gastroesophageal reflux [5], insomnia, body aches [6], anatomical changes including a growing uterus and increased body weight [7], and the addition of a newborn with inconsistent sleep-wake cycles of their own [8]. Some reasons for sleep alterations during this period that are more subtle include hormonal influences [4, 9], sleep disordered breathing [10], restless leg syndrome [11], and changes to sleep architecture itself, which has been shown to change between trimesters [6, 10] and in the post-partum period [1].

Outcomes related to sleep disturbances and changes have been measured in this population primarily using subjective measures, like the Pittsburgh Sleep Quality Index (PSQI) [12]. A recent meta-analysis estimated that 45.7% of pregnant women had poor sleep quality [1] (as defined by PSQI scores > 5) [12]. While commonly used in the research and clinical setting to screen for sleep disorders in pregnant patients, subjective sleep measures must be interpreted with caution as they are generally less accurate than objective measures [13] by overestimating sleep latency [14] and both over and underestimating sleep duration [14, 15].

There are fewer studies assessing objective sleep; typically measured by polysomnography (PSG), which is the “gold standard” [16]. A review of PSG features during pregnancy showed that there are decreases in slow wave sleep (SWS) and rapid eye movement (REM) sleep stages during the third trimester [17]. Although it provides the most accurate measure of sleep, the use of PSG for research or as a screening tool is limited because it is labor intensive and has a high cost [18]. Additionally, a recent review on sleep in pregnancy highlighted the need for more objective measures to assess sleep since it is a current gap in the literature [3]. This review also recommended the need for longitudinal study of these objective measures to understand the full scope of these changes.

Other less invasive and more cost-effective wearable sleep monitors are becoming widely available for the general population [19]. Wearable devices record physiologic data similar to PSG in a less invasive way and can be used outside of the clinical environment for multiple nights. However, limited validation of commercial wearable devices is a barrier to their widespread use in research or clinical settings [20]. These devices have also not yet been substantially studied in the pregnant or post-partum population with regards to sleep data acquisition outside of exploratory studies [21]. One such wearable device from WHOOP (Strap 2.0; WHOOP, Inc., Boston, MA, USA) has been shown to have high concordance with PSG, with bias error ranging from 0.01% on REM sleep duration to 5.5% on non-REM (NREM) sleep duration and precision error ranging 6.69–7.8% respectively [22].

Due to existing evidence that objective and subjective sleep quality may diminish during pregnancy, there is a need to determine what interventions can help to improve sleep quality. There is some existing evidence that physical activity can help improve sleep during pregnancy. A randomized control trial of aquatic physical activity and sleep during the second and third trimester showed significant improvements in self-reported sleep quality among the intervention group [23]. Another indicated that reported moderate levels of physical activity during the first and third trimester were associated with better sleep quality [24]. Other studies specifically suggest that insufficient physical activity is associated with poor sleep quality during pregnancy and that increased exercise in early pregnancy can improve this [25, 26]. A systematic review and meta-analysis on physical activity and sleep during pregnancy indicated that pregnant women who regularly exercised had improved sleep quality [27]. However, to our knowledge, none of these studies explore the association of sleep and physical activity from pre-pregnancy to postpartum while using more objective measures such as wearable devices to measure sleep.

The aim of the present study is to investigate objective changes in sleep pre-pregnancy and throughout the postpartum period as measured by the wearable WHOOP strap. Specifically, our first aim was to use descriptive analyses to examine sleep patterns during the pre-conception, pregnancy, and post-partum time periods in a cohort of healthy women who were physically active at least three days a week prior to conception. Our second aim was to understand the relationship between physical activity during pregnancy and variations in sleep patterns, including awake hours, light sleep hours, deep sleep hours (also called slow wave sleep), and REM sleep hours. We hypothesized that increased activity would be associated with positive changes in sleep patterns during pregnancy and the immediate post-partum period after controlling for pertinent sleep covariates, such as seasonality and weekend sleep patterns.

## Methods

### Recruitment

Following Institutional Review Board approval (#1912819563) at West Virginia University (WVU), a total of 38 women were enrolled following informed written consent from March 2019 until August 2020. Inclusion criteria included women ages 18–35 years old who were currently physically active at least three or more times per week. Currently, ACOG recommends that women who regularly exercised before pregnancy could continue with no adverse effects, which is why individuals had to currently have a moderate level of physical activity to participate [28]. Women who were not currently pregnant, but hoping to conceive within the next six months, were also asked to participate. Prior research on this sample illustrating heart rate variability (HRV) and resting heart rate (RHR) findings during the perinatal period have already been published [29]. Recruitment occurred through the Reproductive Endocrinology and Infertility clinic at WVU and data was collected between March 2019 and July 2021.

### Wearable device

Participants were given a WHOOP^®^ Strap 2.0 (WHOOP, Inc., Boston, MA, USA) and asked to wear it continuously from the time of enrollment and throughout pregnancy, delivery, and postpartum. The WHOOP^®^ strap has reasonable validation with PSG and has been suggested as a practical alternative for that more invasive measure [30]. The WHOOP^®^ devices were purchased with internal research funds through the WVU Center for Reproductive Medicine and provided to participants to keep as an incentive. Participants wore the strap on their nondominant arm allowing it to transmit continuous data to the participant’s smartphone and to a WHOOP cloud platform. Participants were able to see their daily physical activity information on their phones. The comprehensive data from all participants was then downloaded from the WHOOP cloud platform for analysis.

### Measures

Data were imported from WHOOP^®^ in two different tables: (1) daily sleep measures and (2) recorded activity per participant. These tables were then combined with pertinent pregnancy information (i.e., date of conception, date of birth event). More details about variables utilized within this study from these three tables are presented below.

*Daily sleep measures* were assessed using reflectance photoplethysmography and motion with a three-axis accelerometer. That data was then put into algorithms to generate statistics on the participant’s sleep. Daily sleep included four measures: awake hours, light sleep hours, deep sleep hours, and REM sleep hours. Some measures also included a percentage of time asleep in each of those four assessments. These variables have been independently validated against sleep logs, polysomnography, and patient-reported sleep outcomes [22].

*Recorded Daily Activity* was measured by the WHOOP^®^ strap by a three-axis accelerometer and processed using a proprietary algorithm to create daily activity records. Recorded Activities were merged into a day-by-day measure of total time by day spent in each activity, and the three tables were merged by user ID and date. Daily minutes spent in a recorded activity in any zone were included as daily activity minutes, and daily minutes of zone 3 heart rate and higher were converted into a daily moderate/vigorous minutes variable.

*Pregnancy*,* Seasonality*,* and Weekend Timing Information.* These sleep and activity tables were connected to the women’s pertinent pregnancy dates, including conception and delivery date. Season was calculated by stripping the month from the date variable, and recoded as Spring (March-May), Summer (June-August), Fall (September-November), and Winter (December-February). The dates were also used to inform a Weekend/Weekday variable, which was then coded as 1 for Weekday, and 0 for Weekend. After calculating time and date variables, these dates were stripped from the data and not otherwise utilized.

### Data analysis

The longitudinal data was assessed using a linear mixed models approach. After assumptions were checked and found satisfactory, several models were tested, including random intercept, random slope, both random intercept and slope, along with continuous (e.g., day to delivery) and categorical (e.g., trimester) time effects. To constrain time of pregnancy, analyses were restricted to 43 weeks prior to delivery and 8 weeks postpartum; this also ensured that most participants had data for the majority of the time period. Splines were fitted for non-linear patterns including continuous time fixed effects. Models were selected for best fit using lowest Akaike information criteria (AIC). Kuder-Richardson degree of freedom correction was used for all models. Restricted Maximum Likelihood (REML) method was used for all available data, which is appropriate for use with missing at random (MAR) or missing completely at random (MCAR) data. The best fitting model included a random intercept and random continuous time slope (days to delivery), with a variance components covariance matrix and splines set at birth. Two models are presented for each of the four outcomes (awake hours, light sleep hours, deep sleep hours, and REM sleep hours): Model 1: main effects only, including activity minutes per day, days until delivery, days after delivery, season with winter as the referent group, and weekday with weekend as the referent group; Model 2: all main effects in model one, but additionally includes the moderating effects of activity minutes per day on days until delivery and days after delivery. Fixed effects estimates along with standard errors, df, t-value and p-value are presented for each model. Sensitivity analyses included replacing activity minutes with moderate/vigorous activity minutes per day; all effects were similar although slightly attenuated so only the activity minutes models are included here. Additional sensitivity analyses included percentage of time spent in REM and deep sleep. In general, model fit was improved with hours spent in each sleep stage included in the model compared to using the percentage of each sleep stages’ duration. Thus, only models in hours are presented here. All data were analyzed using SAS 9.4 [31]. All descriptive statistics are reported either as frequencies and valid percentages of categorical variables, or mean, standard deviation, minimum and maximum values for continuous variables. For some descriptive analyses, data were summarized by both participant and week of pregnancy.

## Results

### Participants

An initial 38 participants were enrolled in the study, although eight withdrew due to no longer planning to conceive, stated discomfort of the wearable device, or infertility stress. Additionally, a total of 12 participants did not conceive during the study period, leaving birth data for 18 participants. Women were followed for an average of 405.83 days (SD = 153.71; minimum = 142, maximum = 754) with a total of 7,305 days logged. Participants on average took 5.5 months to conceive. Few medical conditions were reported in monthly surveys that the women took, and all participants reported being moderately physically active throughout the perinatal period. All women gave birth between 37 and 41 weeks of pregnancy (39.16 ± 1.26), and their average age at time of delivery was 31.67 years (SD = 2.66). All births in this study were singleton, and the majority were delivered vaginally (61.11%). Further demographics can be found in Table 1.

**Table 1:** Demographics and descriptive statistics for participants (*N* = 18)

Variable	M or *n* (SD or %)
**Age at delivery**	31.67 (2.66)
**Prior pregnancy**	
0	9 (50.00)
1–2	9 (50.00)
**Prior live birth**	
Yes	4 (22.22)
No	14 (77.78)
**Medical Complications in Pregnancy** ^**a**^	
Yes	1 (5.56)
No	17 (94.44)
**Medical Complications in Birth** ^**b**^	
Yes	5 (27.78)
No	13 (72.22)
Mode of Delivery*	
Vaginal (or VBAC)	10 (55.56)
C-section	7 (38.89)
**Weeks at Delivery**	39.16 (1.26)
Birth Weight*	7.24 (0.91) lbs.

^*^ Missing information on 1 participant, ^a^ e.g.: Gestational hypertension, ^b^ e.g.: Postpartum hemorrhage

### Changes during pregnancy

Based on WHOOP^®^ data, activity levels decreased over the course of the pregnancy from almost 28 min of logged daily activity pre-pregnancy to 14 min by the third trimester (Table 2), with a slight increase during post-partum (almost 16 min). There was a similar pattern for time spent in deep sleep (in hours) by trimester (decreased from 1.4 h to 1.2 h, and then a slight improvement to 1.3 h during post-partum period). Inversely, the number of awake hours (during sleep periods) steadily increased throughout the entire study (from 0.65 h to 1.17 h). REM sleep hours steadily increased until birth (from 1.6 to 2.0) with a decrease post-partum that was still higher than pre-pregnancy amounts (1.8). Light sleep hours were more variable, increasing from pre-pregnancy (4.0) to first trimester (4.2) and then decreasing steadily to post-partum rates (3.4).

**Table 2:** Descriptive statistics for variables of interest; *n* = 18 across 7,305 days of data

Variable	M (SD)	Pre-pregnancy M (SD) *n* = 1796	First Trimester M (SD) *n* = 1225	Second Trimester M (SD) *n* = 1582	Third Trimester M (SD) *n* = 1072	Post-partum M (SD) *n* = 1630
Hours of Sleep	6.97 (1.59)	7.08 (1.34)	7.25 (1.47)	7.24 (1.49)	6.81 (1.58)	6.47 (1.87)
Awake hours	0.85 (0.75)	0.65 (0.64)	0.68 (0.52)	0.77 (0.62)	0.98 (0.74)	1.17 (0.98)
Deep sleep hours	1.33 (0.45)	1.44 (0.43)	1.34 (0.41)	1.33 (0.41)	1.20 (0.46)	1.30 (0.48)
Deep Sleep %	17.15 (4.87)	18.59 (4.59)	17.04 (4.45)	16.69 (4.54)	15.33 (5.16)	17.31 (5.10)
REM sleep hours	1.81 (0.81)	1.62 (0.71)	1.73 (0.78)	1.94 (0.83)	2.00 (0.85)	1.82 (0.82)
REM Sleep %	23.10 (9.01)	20.88 (8.40)	21.89 (8.91)	24.13 (8.94)	25.51 (8.87)	23.85 (9.26)
Light sleep hours	3.83 (1.17)	4.03 (1.07)	4.17 (1.17)	3.97 (1.11)	3.61 (1.07)	3.36 (1.23)
Activity minutes - day	20.70 (31.84)	27.44 (34.27)	21.95 (33.35)	18.65 (32.64)	13.68 (25.87)	15.65 (27.59)
Activity minutes, moderate/vigorous - day	13.14 (21.19)	18.15 (24.94)	14.05 (23.07)	10.73 (17.90)	7.61 (14.60)	9.83 (17.70)

### Model results

The linear mixed model results are presented for awake hours (Table 3), light sleep hours (Table 4), deep sleep hours (Table 5), and REM sleep hours (Table 6). Both main effects models (Model 1) and the adjusted model controlling for the moderating effect of activity minutes (Model 2) are included in all tables. The presented results focus on interpretation of the moderating effects models given improved model fit.

**Table 3:** Fixed effects parameter estimates from linear mixed models for awake hours (*n* = 18). Model 1: main effects. Model 2: moderating effects of activity minutes/day

Independent Variable	Estimate	SE Estimate	Df	t-value	*p*-value
Model 1: Main effects					
Intercept	1.13	0.13	18.3	8.89	< 0.0001
Activity (minutes/day)	0.0002	0.0003	4926	0.63	0.53
Days until delivery	0.002	0.0005	17.2	4.42	0.0004
Days post-delivery	0.008	0.001	4945	7.35	< 0.0001
*Season (ref = Winter)*					
Fall	0.05	0.03	4822	1.66	0.10
Spring	0.06	0.03	4849	2.18	0.03
Summer	0.11	0.03	4361	3.35	0.0008
*Weekday (ref = No)*					
Yes	-0.05	0.02	4911	-2.33	0.02
**Model 2: Main effects plus moderating activity minutes**					
Intercept	1.11	0.13	18.6	8.79	< 0.0001
Activity (minutes/day)	0.0009	0.0008	4934	1.24	0.22
Days until delivery	0.002	0.0005	17.9	4.29	0.0004
Days post-delivery	0.010	0.001	4942	7.66	< 0.0001
*Season (ref = Winter)*					
Fall	0.05	0.03	4813	1.63	0.10
Spring	0.07	0.03	4844	2.29	0.02
Summer	0.11	0.03	4344	3.41	0.0007
*Weekday (ref = No)*					
Yes	-0.05	0.02	4909	-2.27	0.02
*Moderators*					
Activity *Days until delivery	0.000	0.000	4933	0.77	0.44
Activity *Days post-delivery	-0.0001	0.000	4922	-2.75	0.006

*Abbreviations: ref: Reference group

**Table 4:** Fixed effects parameter estimates from linear mixed models for light sleep (*n* = 18). Model 1: main effects. Model 2: moderating effects of activity minutes/day

Independent Variable	Estimate	SE Estimate	Df	t-value	*p*-value
Model 1: Main effects					
Intercept	3.35	0.16	17.8	20.47	< 0.0001
Activity (minutes/day)	0.0003	0.0005	4930	0.57	0.57
Days until delivery	-0.004	0.0007	17.4	-5.13	< 0.0001
Days post-delivery	-0.005	0.002	4943	-2.78	0.006
*Season (ref = Winter)*					
Fall	0.09	0.05	4698	2.08	0.04
Spring	0.16	0.04	4750	3.46	0.0005
Summer	-0.09	0.05	3959	-1.81	0.07
*Weekday (ref = No)*					
Yes	-0.07	0.03	4910	-2.29	0.02
**Model 2: Main effects plus moderating activity minutes**					
Intercept	3.29	0.17	18.2	19.83	< 0.0001
Activity (minutes/day)	0.004	0.001	4940	3.78	0.0002
Days until delivery	-0.004	0.0007	18.4	-5.69	< 0.0001
Days post-delivery	-0.003	0.002	4944	-1.64	0.10
*Season (ref = Winter)*					
Fall	0.08	0.05	4700	1.86	0.06
Spring	0.16	0.05	4755	3.55	0.0004
Summer	-0.09	0.05	3980	-1.79	0.07
*Weekday (ref = No)*					
Yes	-0.07	0.03	4908	-2.26	0.02
*Moderators*					
Activity *Days until delivery	0.00003	0.000	4938	4.04	< 0.0001
Activity *Days post-delivery	-0.00008	0.00006	4926	-1.29	0.20

*Abbreviations: ref: Reference group

**Table 5:** Fixed effects parameter estimates from linear mixed models for deep sleep (*n* = 18). Model 1: main effects. Model 2: moderating effects of activity minutes/day

Independent Variable	Estimate	SE Estimate	Df	t-value	*p*-value
Model 1: Main effects					
Intercept	1.12	0.05	18	20.97	< 0.0001
Activity (minutes/day)	0.0005	0.0002	4940	2.45	0.014
Days until delivery	-0.001	0.0002	14.4	-7.81	< 0.0001
Days post-delivery	0.004	0.0007	4727	5.5	< 0.0001
*Season (ref = Winter)*					
Fall	0.05	0.018	2848	2.86	0.004
Spring	-0.03	0.01	3167	-1.71	0.09
Summer	0.005	0.02	1286	0.27	0.79
*Weekday (ref = No)*					
Yes	-0.05	0.01	4897	-3.84	0.0001
**Model 2: Main effects plus moderating activity minutes**					
Intercept	1.10	0.05	18.6	20.46	< 0.0001
Activity (minutes/day)	0.002	0.0005	4918	3.41	0.0006
Days until delivery	-0.001	0.0002	17.1	-8.20	< 0.0001
Days post-delivery	0.004	0.0008	4810	5.32	< 0.0001
*Season (ref = Winter)*					
Fall	0.05	0.02	2866	2.72	0.007
Spring	-0.03	0.02	3209	-1.63	0.10
Summer	0.006	0.02	1316	0.30	0.76
*Weekday (ref = No)*					
Yes	-0.05	0.01	4896	-3.81	0.0001
*Moderators*					
Activity *Days until delivery	0.0000	0.000	4894	2.64	0.008
Activity *Days post-delivery	-0.00003	0.00002	4937	-1.36	0.17

*Abbreviations: ref: Reference group

**Table 6:** Fixed effects parameter estimates from linear mixed models for REM sleep (*n* = 18). Model 1: main effects. Model 2: moderating effects of activity minutes/day

Independent Variable	Estimate	SE Estimate	Df	t-value	*p*-value
Model 1: Main effects					
Intercept	2.06	0.11	19.2	18.47	< 0.0001
Activity (minutes/day)	0.0007	0.0003	4936	2.29	0.02
Days until delivery	0.0006	0.0003	18.7	1.98	0.06
Days post-delivery	-0.007	0.001	4898	-6.24	< 0.0001
*Season (ref = Winter)*					
Fall	-0.17	0.03	4142	-5.71	< 0.0001
Spring	-0.004	0.03	4290	-0.13	0.89
Summer	-0.09	0.03	2787	-2.90	0.004
*Weekday (ref = No)*					
Yes	-0.13	0.02	4915	-6.19	< 0.0001
**Model 2: Main effects plus moderating activity minutes**					
Intercept	2.01	0.11	19.6	17.96	< 0.0001
Activity (minutes/day)	0.005	0.0008	4941	5.93	< 0.0001
Days until delivery	0.0002	0.0003	20.6	0.73	0.47
Days post-delivery	-0.005	0.001	4916	-3.72	0.0002
*Season (ref = Winter)*					
Fall	-0.18	0.03	4159	-6.02	< 0.0001
Spring	0.001	0.03	4316	0.03	0.97
Summer	-0.09	0.03	2835	-2.85	0.004
*Weekday (ref = No)*					
Yes	-0.13	0.02	4913	-6.13	< 0.0001
*Moderators*					
Activity *Days until delivery	0.000	0.000	4936	5.52	< 0.0001
Activity *Days post-delivery	-0.0001	0.000	4933	-2.77	0.005

*Abbreviations: ref: Reference group

**Awake hours.** Consistent with descriptive results, we found an increase in awake hours both during pregnancy (Est. = 0.002, *p* = 0.0004) and post-partum (Est. = 0.01, *p* < 0.0001). This means that per day of pregnancy, there was a 0.002 increase in the number of awake hours during sleep. Both Spring (Est.=0.07, *p* = 0.02) and Summer (Est.=0.011, *p* = 0.0007) were associated with an increased time in awake hours relative to winter. Additionally, during the weekday, women spent less time awake relative to the weekend (Est. = -0.05, *p* = 0.02). No moderating effect of physical activity was found for awake hours during pregnancy; however, increased physical activity in minutes post-partum demonstrated a significant decrease of awake time (Est. = -0.001, *p* = 0.006).

**Light sleep hours.** More physical activity minutes were associated with an increase in light sleep (Est. = 0.004, *p* = 0.0002). Light sleep hours generally decreased across pregnancy (Est. = -0.004, *p* < 0.0001). There was an increase in light sleep hours during the Spring relative to Winter (Est. = 0.16, *p* = 0.0004) and a decrease on weekdays relative to weekends (Est. = -0.07, *p* = 0.02). More physical activity minutes during pregnancy increased light sleep hours over the course of the pregnancy (Est. = 0.00003, *p* < 0.0001).

**Deep sleep hours**. More physical activity minutes were associated with an increase in deep sleep (Est. = 0.002, *p* = 0.0006). Deep sleep hours generally decreased across pregnancy (Est. = -0.001, *p* < 0.0001) but improved post-partum (Est.=0.004, *p* < 0.0001). There was a decrease in deep sleep hours on weekdays relative to weekends (Est. = -0.05, *p* = 0.0001). More physical activity minutes during pregnancy were associated with increased deep sleep hours over the course of the pregnancy (Est. = 0.000001, *p* = 0.008).

**REM sleep hours**. More physical activity minutes were also associated with an increase in REM sleep (Est. = 0.005, *p* < 0.0001). REM sleep hours rapidly decreased post-partum (Est. = -0.005, *p* = 0.0002). A decrease in REM sleep was experienced during the Fall (Est.=-0.18, *p* < 0.0001) and Summer (Est. = -0.09, *p* = 0.004) relative to Winter; and a decrease in REM sleep hours on Weekdays relative to Weekends (Est. = -0.13, *p* < 0.0001). More physical activity minutes during pregnancy increased REM sleep hours over the course of the pregnancy (Est. = 0.000001, *p* < 0.0001); however, more physical activity minutes during post-partum appeared to decrease REM sleep hours (Est. = -0.0001, *p* = 0.00).

## Discussion

The cohort of 18 women demonstrated continued physical activity throughout pregnancy and following their delivery. Activity minutes (of any intensity) and vigorous activity minutes decreased in the third trimester and then increased again postpartum. Sleep duration was just below 7 h per night (M = 6.97, SD = 1.59) and decreased in the third trimester and during postpartum. These results show some interruptions to both sleep and exercise in the final trimester and following birth, which is expected based on physiological changes to prepare for birth, as well as lifestyle changes following birth.

REM sleep when measured with the PSG typically has been found to decrease during pregnancy [32]. Instead, REM sleep typically transitions to more NREM sleep closer to birth. Our findings suggest that REM sleep slightly increases closer to birth, in contradiction with previous findings. More research is needed to understand these differences, some of which could be due to measurement type (PSG vs. WHOOP).

The overall findings indicate that moderate to vigorous physical activity minutes were associated with overall deep sleep hours during pregnancy, but not in postpartum. Similar effects were found for all activity minutes, regardless of intensity, although moderation by physical activity minutes was no longer significant postpartum.

However, it is also of note that these women were not meeting the guidelines of 150 min of moderate to vigorous physical activity (MVPA) per week. The average minutes of MVPA per day was about 13 min per day, totalling on average, 91 min per week. In this area of Appalachia, however, only 46.8% of women meet the physical activity guidelines of 150 min of physical activity per week [33]. Other research has suggested only 17.7% of pregnant women meet these guidelines [34]. So, although these rates of MVPA physical activity are not at the recommended levels, they are consistent with what is found in this population.

Additionally, although the number of physical activity minutes, especially later in pregnancy, was quite low, this could still have a meaningful effect on sleep and health. A recent study indicated that it was the number of steps per day (or overall physical activity) rather than MVPA that was associated with cardiovascular health [35]. Additionally, women’s overall sleep percentage increased by.09 extra points when they did light exercise in the afternoon. Another study comparing moderate aerobic exercise to usual prenatal care found improvements in all areas of sleep quality, although these were not statistically significant [36]. This also indicates that even when the effect of physical activity may be lower, there is still clinical and meaningful significance to the individual.

These results corroborate previous studies and meta-analyses indicating that physical activity may be associated with sleep quality [23–25, 27]. Additionally, this study extends the research regarding this topic by providing evidence of how physical activity affects sleep pre-conception as well as during postpartum.

Results suggest that any type of activity as well as moderate to vigorous activity is associated with improved sleep quality across the perinatal period. The implications of these findings provide greater understanding to both clinicians as well as their patients about the multi-faceted importance of remaining physically active throughout the perinatal period. It also helps empower pregnant women by understanding that any level of physical activity might help, which assists with motivation if individuals are less willing or able to undertake higher intensity exercise.

Additionally, because this sample includes some women pursuing and conceiving through assisted reproductive technology (ART), the conclusions from this study may be clinically useful in improving fertility outcomes. Research has shown that sleep duration and quality can be a modifiable target to increase the number of oocytes retrieved during a fertility cycle [37]. Other research indicates that poor sleep quality contributes to psychological distress for couples pursuing ART and that it can affect pregnancy outcomes [38]. Therefore, understanding the ways in which physical activity is associated with improved sleep may indirectly contribute to better fertility outcomes for this population throughout the perinatal period. The use of wearable technology for these issues among individuals pursuing ART (and non-ART samples) can provide individuals and their clinicians with more immediate data with which to modify physical activity and sleep quality.

### Strengths, limitations, & future directions

This study has several strengths. First, although there are only 18 participants included in this study, a substantial amount of data was obtained for each participant due to continuous monitoring by the WHOOP^®^ straps, which provided an adequate data set for analysis. Second, the WHOOP^®^ strap resulted in daily readings showing patterns for sleep and exercise for each day of pregnancy. Third, we followed women throughout the perinatal period, allowing for a more in-depth look into sleep and exercise changes throughout that time. Fourth, this wearable device is a more objective measure of sleep than other studies, which have used the PSQI.

Some women included in the analysis were seeking fertility treatment, which limits the generalizability of the study to other pregnant women. Additionally, the WHOOP^®^ strap is not quite as accurate as clinical measures, but still provides meaningful data regarding sleep and other indicators. Another limitation is that we cannot determine directionality (causality) for the changes in the variables. Although physical activity was found to be associated with improved sleep, it could be that individuals with improved sleep are more able to engage in physical activity. Additionally, since the WHOOP^®^ strap is worn on the wrist, it cannot measure body position. The WHOOP^®^ strap, although validated against the PSG [30], has not currently been validated during pregnancy, which is needed to fully understand how it performs in this population. Finally, the act of tracking behaviors and seeing results in real time may contribute to behavior change; therefore, some changes to physical activity and/or sleep may have been related to tracking behaviors [39].

Future research will focus on larger samples, as well as using additional measures to assess body position and posture. There is burgeoning research to suggest that sleeping posture during the perinatal period can be associated with placental location [40]. More work also needs to be done to validate the WHOOP strap against the PSG in pregnancy. Understanding sleep duration, quality, and position could provide more insight into maternal and child health and outcomes.

## Conclusion

Wearable technology, such as WHOOP^®^ (Strap 2.0; WHOOP, Inc., Boston, MA, USA) straps, may provide a convenient method for measuring sleep in comparison to more invasive or expensive assessments. This type of wearable technology can be particularly useful for a pregnant and postpartum population to monitor both sleep and activity levels during the perinatal period. The ability for individuals and clinicians to monitor and improve physical activity and sleep across the perinatal period can be helpful on a broad level, but also in a targeted way for those who may be using ART. Moderating effects suggest positive health influences of both moderate-vigorous and any activity minutes during pregnancy and post-partum on sleep outcomes. These results suggest important clinical implications to assist with improving maternal sleep outcomes during the perinatal period, by encouraging physical activity.

## Data Availability

Data is available upon reasonable request.

## Abbreviations

AIC: Akaike information criteria
ART: Assisted reproductive technology
HRV: Heart rate variability
MAR: Missing at random
MCAR: Missing completely at random
MVPA: Moderate to vigorous physical activity
PSQI: Pittsburgh Sleep Quality Index
PSG: Polysomnography
REM: Rapid eye movement
RHR: Resting heart rate
REML: Restricted Maximum Likelihood
SWS: Slow wave sleep
WVU: West Virginia University

## References

[1] Sedov ID, Cameron EE, Madigan S, Tomfohr-Madsen LM. Sleep quality during pregnancy: A meta-analysis. *Sleep Medicine Reviews*. 2018/04/01 2018;38:168–176. doi:10.1016/j.smrv.2017.06.005. 10.1016/j.smrv.2017.06.005 28866020

[2] Reid KJ, Facco FL, Grobman WA, et al. Sleep during pregnancy: the nuMoM2b pregnancy and sleep duration and continuity study. Sleep May. 2017;1(5). 10.1093/sleep/zsx045. 10.1093/sleep/zsx045 PMC6396817 28369543

[3] Kember AJ, Elangainesan P, Ferraro ZM, Jones C, Hobson SR. Common sleep disorders in pregnancy: a review. Front Med (Lausanne). 2023;10:1235252. 10.3389/fmed.2023.1235252. 37671402 10.3389/fmed.2023.1235252 PMC10475609

[4] Santiago JR, Nolledo MS, Kinzler W, Santiago TV. Sleep and Sleep Disorders in Pregnancy. *Annals of Internal Medicine*. 2001/03/06 2001;134(5):396–408. 10.7326/0003-4819-134-5-200103060-00012 10.7326/0003-4819-134-5-200103060-00012 11242500

[5] Habr F, Raker C, Lin CL, Zouein E, Bourjeily G. Predictors of gastroesophageal reflux symptoms in pregnant women screened for sleep disordered breathing: a secondary analysis. Clin Res Hepatol Gastroenterol. 2013;2013/02/01(1):93–9. 10.1016/j.clinre.2012.03.036. 10.1016/j.clinre.2012.03.036 22572522

[6] Hertz G, Fast A, Feinsilver SH, Albertario CL, Schulman H, Fein AM. Sleep in Normal Late Pregnancy. Sleep. 1992/05/01 1992;15(3):246–251. doi:10.1093/sleep/15.3.246. 10.1093/sleep/15.3.246 1621025

[7] Pengo MF, Won CH, Bourjeily G. Sleep in women across the Life Span. Chest. 2018;154(1):196–206. 10.1016/j.chest.2018.04.005. 07/01 2018. 29679598 10.1016/j.chest.2018.04.005 PMC6045782

[8] Lee KA. Alterations in sleep during pregnancy and postpartum: a review of 30 years of research. Sleep Med Rev. 1998;11(4):231–42. 10.1016/S1087-0792(98)90010-7. /01 1998. 10.1016/s1087-0792(98)90010-7 15310494

[9] Manber R, Armitage R. Sex, Steroids, and Sleep: A Review. Sleep. 1999/08/01 1999;22(5):540–541. 10.1093/sleep/22.5.540 10450590

[10] Pien GW, Schwab RJ. Sleep disorders during pregnancy. Sleep. 2004;27(7):1405–17. 10.1093/sleep/27.7.1405. 10/01 2004. 15586794 10.1093/sleep/27.7.1405

[11] Facco FL, Kramer J, Ho KH, Zee PC, Grobman WA. Sleep Disturbances in Pregnancy. *Obstetrics & Gynecology*. 2010/01 2010;115(1):77–83. 10.1097/AOG.0b013e3181c4f8ec 10.1097/AOG.0b013e3181c4f8ec 20027038

[12] Buysse DJ, Reynolds CF, Monk TH, Berman SR, Kupfer DJ. The Pittsburgh sleep quality index: A new instrument for psychiatric practice and research. Psychiatry Research. 1989/05/01 1989;28(2):193–213. doi: 10.1016/0165-1781(89)90047-4. 10.1016/0165-1781(89)90047-4 2748771

[13] Okun ML, Kohl V, Feliciano L. Comparison of longitudinal diary and actigraphy-assessed sleep in pregnant women. Sleep Medicine. 2021/12/01 2021;88:149–156. doi: 10.1016/j.sleep.2021.09.015. 10.1016/j.sleep.2021.09.015 34753041

[14] Wilson DL, Fung A, Walker SP, Barnes M. Subjective Reports Versus Objective Measurement of Sleep Latency and Sleep Duration in Pregnancy. *Behavioral Sleep Medicine*. 2013/07/01 2013;11(3):207–221. 10.1080/15402002.2012.670674 10.1080/15402002.2012.670674 23205562

[15] Herring SJ, Foster GD, Pien GW et al. Do pregnant women accurately report sleep time? A comparison between self-reported and objective measures of sleep duration in pregnancy among a sample of urban mothers. *Sleep and Breathing*. 2013/12/01 2013;17(4):1323–1327. 10.1007/s11325-013-0835-2 10.1007/s11325-013-0835-2 PMC3796189 23563909

[16] Rundo JV, Downey R. Chapter 25 - Polysomnography. In: Levin KH, Chauvel P, Levin KH, Chauvel P, eds. *Handbook of Clinical Neurology*. 2019:381–392. *Clinical Neurophysiology: Basis and Technical Aspects*. 10.1016/B978-0-444-64032-1.00025-4 31277862

[17] Garbazza C, Hackethal S, Riccardi S et al. Polysomnographic features of pregnancy: a systematic review. Sleep Med Rev. 2020/04/01 2020;50:101249. 10.1016/j.smrv.2019.101249 10.1016/j.smrv.2019.101249 31896508

[18] Zhu B, Calvo RS, Wu L, et al. Objective sleep in pregnant women: a comparison of actigraphy and polysomnography. Sleep Health. 2018;4(5):390–6. 10.1016/j.sleh.2018.07.011. 2018/10. 30241652 10.1016/j.sleh.2018.07.011 PMC6204346

[19] Kwon S, Kim H, Yeo W-H. Recent advances in wearable sensors and portable electronics for sleep monitoring. iScience. 2021/05/21 2021;24(5):102461. 10.1016/j.isci.2021.102461 10.1016/j.isci.2021.102461 PMC8113882 34013173

[20] Guillodo E, Lemey C, Simonnet M et al. Clinical applications of Mobile Health Wearable–Based Sleep monitoring: systematic review. JMIR mHealth uHealth. 2020/04/01 2020;8(4):e10733. 10.2196/10733 10.2196/10733 PMC7160700 32234707

[21] Nulty AK, Chen E, Thompson AL. The Ava bracelet for collection of fertility and pregnancy data in free-living conditions: An exploratory validity and acceptability study. *DIGITAL HEALTH*. 2022/01/01 2022;8:20552076221084461. 10.1177/20552076221084461 10.1177/20552076221084461 PMC8918962 35295766

[22] Berryhill S, Morton CJ, Dean A, et al. Effect of wearables on sleep in healthy individuals: a randomized crossover trial and validation study. J Clin Sleep Med May. 2020;15(5):775–83. 10.5664/jcsm.8356. 10.5664/jcsm.8356 PMC7849816 32043961

[23] Rodriguez-Blanque R, Sánchez-García JC, Sánchez-López AM, Mur-Villar N, Aguilar-Cordero MJ. The influence of physical activity in water on sleep quality in pregnant women: A randomised trial. *Women and Birth*. 2018/02/01/ 2018;31(1):e51-e58. 10.1016/j.wombi.2017.06.018 10.1016/j.wombi.2017.06.018 28693969

[24] Tan L, Zou J, Zhang Y, Yang Q, Shi H. A longitudinal study of physical activity to improve Sleep Quality during pregnancy. Nat Sci Sleep. 2020;12:431–42. 10.2147/nss.S253213. 32765140 10.2147/NSS.S253213 PMC7367923

[25] Du M, Liu J, Han N et al. Maternal sleep quality during early pregnancy, risk factors and its impact on pregnancy outcomes: a prospective cohort study. Sleep Medicine. 2021/03/01 2021;79:11–18. doi: 10.1016/j.sleep.2020.12.040. 10.1016/j.sleep.2020.12.040 33454523

[26] Baker JH, Rothenberger SD, Kline CE, Okun ML. Exercise during early pregnancy is associated with greater sleep continuity. Behav Sleep Med. 2018;09(5):482–93. 10.1080/15402002.2016.1228649. /03 2018. 10.1080/15402002.2016.1228649 PMC6124311 27739877

[27] Yang S-Y, Lan S-J, Yen Y-Y, Hsieh Y-P, Kung P-T, Lan S-H. Effects of Exercise on Sleep Quality in pregnant women: a systematic review and Meta-analysis of Randomized controlled trials. Asian Nurs Res. 2020;2020/02/01(1):1–10. 10.1016/j.anr.2020.01.003. 10.1016/j.anr.2020.01.003 32006719

[28] Physical Activity and Exercise During Pregnancy and the Postpartum Period. ACOG Committee Opinion, Number 804. Obstet Gynecol Apr. 2020;135(4):e178–88. 10.1097/aog.0000000000003772. 10.1097/AOG.0000000000003772 32217980

[29] Rowan SP, Lilly CL, Claydon EA, Wallace J, Merryman K. Monitoring one heart to help two: heart rate variability and resting heart rate using wearable technology in active women across the perinatal period. *BMC Pregnancy and Childbirth*. 2022/11/30 2022;22(1):887. 10.1186/s12884-022-05183-z 10.1186/s12884-022-05183-z PMC9710029 36451120

[30] Miller DJ, Lastella M, Scanlan AT, et al. A validation study of the WHOOP strap against polysomnography to assess sleep. J Sports Sci Nov. 2020;38(22):2631–6. 10.1080/02640414.2020.1797448. 10.1080/02640414.2020.1797448 32713257

[31] SAS Institute Inc. SAS/ACCESS(R) 9.4 interface to ADABAS:reference. SAS Institute Inc.; 2013.

[32] Garbazza C, Hackethal S, Riccardi S et al. Apr. Polysomnographic features of pregnancy: A systematic review. *Sleep Medicine Reviews*. 2020;50, doi:ARTN 101249 10.1016/j.smrv.2019.101249 10.1016/j.smrv.2019.101249 31896508

[33] Abildso CG, Daily SM, Meyer MRU, et al. Environmental Factors Associated with Physical Activity in Rural U.S. counties. Int J Environ Res Public Health Jul. 2021;20(14). 10.3390/ijerph18147688. 10.3390/ijerph18147688 PMC8307667 34300138

[34] Reynolds LJ, Twiddy HM, Mlynarczyk M, Wilson PB. The association of physical activity on homocysteine in pregnant women. J Matern Fetal Neonatal Med Dec. 2022;35(25):7073–80. 10.1080/14767058.2021.1941855. 10.1080/14767058.2021.1941855 34162283

[35] Wendt A, da Silva ICM, Goncalves H, Menezes A, Barros F, Wehrmeister FC. Short-term effect of physical activity on sleep health: a population-based study using accelerometry. J Sport Health Sci Sep. 2022;11(5):630–8. 10.1016/j.jshs.2020.04.007. 10.1016/j.jshs.2020.04.007 PMC9532894 32422346

[36] Navas A, Carrascosa MDC, Artigues C, et al. Effectiveness of moderate-intensity Aerobic Water Exercise during pregnancy on quality of life and Postpartum Depression: a Multi-center, Randomized Controlled Trial. J Clin Med May. 2021;30(11). 10.3390/jcm10112432. 10.3390/jcm10112432 PMC8198819 34070842

[37] Goldstein CA, Smith YR, Sleep. Circadian rhythms, and fertility. Curr Sleep Med Rep. 2016;12(4):206–17. 10.1007/s40675-016-0057-9. /01 2016.

[38] Philipsen MT, Knudsen UB, Zachariae R, Ingerslev HJ, Hvidt JEM, Frederiksen Y. Sleep, psychological distress, and clinical pregnancy outcome in women and their partners undergoing in vitro or intracytoplasmic sperm injection fertility treatment. Sleep Health Apr. 2022;8(2):242–8. 10.1016/j.sleh.2021.10.011. 10.1016/j.sleh.2021.10.011 34949542

[39] Felder JN, Mirchandaney R, Harrison J et al. Examining Experiences of Poor Sleep During Pregnancy: A Qualitative Study to Inform the Development of a Prenatal Sleep Intervention. Global Advances in Health and Medicine. 2022/12/01 2022;11:2164957X221087655. 10.1177/2164957X221087655 10.1177/2164957X221087655 PMC8961353 35360508

[40] Kember AJ, Anderson JL, House SC, Reuter DG, Goergen CJ, Hobson SR. Impact of maternal posture on fetal physiology in human pregnancy: a narrative review. Front Physiol. 2024;15:1394707. 10.3389/fphys.2024.1394707. 38827993 10.3389/fphys.2024.1394707 PMC11140392

